# Highly specific fiber optic immunosensor coupled with immunomagnetic separation for detection of low levels of *Listeria monocytogenes* and *L. ivanovii*

**DOI:** 10.1186/1471-2180-12-275

**Published:** 2012-11-23

**Authors:** Marcelo Mendonça, Neida L Conrad, Fabricio R Conceição, Ângela N Moreira, Wladimir P da Silva, José AG Aleixo, Arun K Bhunia

**Affiliations:** 1 Laboratório de Imunologia Aplicada, Núcleo de Biotecnologia, Centro de Desenvolvimento Tecnológico, Universidade Federal de Pelotas, 96010-900 Pelotas, RS, Brazil; 2 Laboratório de Microbiologia de Alimentos, Departamento de Ciência e Tecnologia Agroindustrial, Faculdade de Agronomia Eliseu Maciel, Universidade Federal de Pelotas, 96010-900, Pelotas, RS, Brazil; 3 Molecular Food Microbiology Laboratory, Department of Food Science, Purdue University, 745 Agriculture Mall Drive, West Lafayette, IN 47907, USA

**Keywords:** *Listeria monocytogenes*, Internalin A, Monoclonal antibody, Immunomagnetic separation, Fiber optic sensor, Light scattering sensor, qPCR, Detection, Biosensor

## Abstract

**Background:**

Immunomagnetic separation (IMS) and immunoassays are widely used for pathogen detection. However, novel technology platforms with highly selective antibodies are essential to improve detection sensitivity, specificity and performance. In this study, monoclonal antibodies (MAbs) against Internalin A (InlA) and p30 were generated and used on paramagnetic beads of varying diameters for concentration, as well as on fiber-optic sensor for detection.

**Results:**

Anti-InlA MAb-2D12 (IgG2a subclass) was specific for *Listeria monocytogenes* and *L. ivanovii*, and p30-specific MAb-3F8 (IgM) was specific for the genus *Listeria*. At all bacterial concentrations (10^3^–10^8^ CFU/mL) tested in the IMS assay; the 1-μm diameter MyOne beads had significantly higher capture efficiency (*P* < 0.05) than the 2.8-μm diameter M-280 beads with both antibodies. The highest capture efficiency for MyOne-2D12 (49.2% for 10^5^ CFU/mL) was significantly higher (*P* < 0.05) than that of MyOne-3F8 (16.6 %) and Dynabeads anti-*Listeria* antibody (9 *%*). Furthermore, capture efficiency for MyOne-2D12 was highly specific for *L. monocytogenes* and *L. ivanovii*. Subsequently, we captured *L. monocytogenes* by MyOne-2D12 and MyOne-3F8 from hotdogs inoculated with mono- or co-cultures of *L. monocytogenes* and *L. innocua* (10–40 CFU/g), enriched for 18 h and detected by fiber-optic sensor and confirmed by plating, light-scattering, and qPCR assays. The detection limit for *L. monocytogenes* and *L. ivanovii* by the fiber-optic immunosensor was 3 × 10^2^ CFU/mL using MAb-2D12 as capture and reporter antibody. Selective media plating, light-scattering, and qPCR assays confirmed the IMS and fiber-optic results.

**Conclusions:**

IMS coupled with a fiber-optic sensor using anti-InlA MAb is highly specific for *L. monocytogenes* and *L. ivanovii* and enabled detection of these pathogens at low levels from buffer or food.

## Background

The foodborne pathogen *Listeria monocytogenes* causes listeriosis—a severe illness that ranges from mild gastroenteritis to invasive infection in immunocompromised people, neonates, and the elderly [1]. In pregnant women, it causes premature births, miscarriages, and neonatal sepsis or fetal deaths. *L. monocytogenes* is ubiquitous and found in food-processing environments [2,3] and food products, including ethnic soft cheese [4,5], sliced lunch meats [6] and frankfurters, and seafood [7]. It has been implicated in numerous food outbreaks and recalls, including a large outbreak involving cantaloupe in the US, which caused 29 deaths and 1 miscarriage [8]. Listeriosis has an estimated 19% fatality rate and ranks third among all fatalities resulting from foodborne infections in the USA [9]. Therefore, many countries have established a “zero tolerance” policy towards *L. monocytogenes* in RTE foods [10]. Food recalls have increased each year, placing an economic burden on food manufacturers and growers. Rapid and accurate detection methods may alleviate some of these problems.

The genus *Listeria* consists of 8 species: *L. monocytogenes*, *L. ivanovii*, *L. seeligeri*, *L. welshimeri*, *L. innocua*, *L. grayi*, and two new species, *L. marthii*[11] and *L. rocourtiae*[12]. *L. monocytogenes* and *L. ivanovii* are pathogenic to humans and animals [13]. Many virulence and structural genes or gene products in *Listeria* could be used as targets for antibody- or nucleic acid-based assay development [14]. *L. monocytogenes* expresses several virulence proteins [15], including Internalin A (InlA), which promotes bacterial adhesion and invasion of the host cell [15]. InlA possesses N-terminal leucine-rich repeats that facilitate anchoring to the bacterial cell wall, while the most distal extracellular domain binds to E-cadherin, which is crucial for host cell–cell adhesion and maintenance of tissue architecture.

Both pathogenic and non-pathogenic *Listeria* species can be found in the same environment or food [16]. However, when an enrichment step is used, the non-pathogenic species may overgrow and outcompete *L. monocytogenes*[17-19], leading to false-negative results. *L. innocua* is the most frequently found bacteria in *Listeria*-contaminated foods [17,20], thus presenting a challenge for the specific capture and detection of pathogenic *Listeria*[21]. Hence, it is essential to develop methods that are capable of detecting pathogenic species in the presence of non-pathogenic species.

Immunological approaches to detect pathogens in food are attractive; however, assay performance depends on the quality and specificity of the antibodies used [14,22]. For detection of *Listeria*, two types of assay specificities are desired: *Listeria* genus- or *L. monocytogenes*-specific tests. Anti-*Listeria* antibodies available from research laboratories or commercial vendors are associated with problems of low affinity [23], reaction to heterologous antigens [24,25], lack of reaction towards all serotypes of *L. monocytogenes*[23,26-28], lack of reaction due to physiological stress induced by growth media or assay parameters [29,30], and lack of compatibility with certain bioassay platforms [14,22,31]. Thus, there is a need for continued efforts to produce high-quality antibodies.

The recovery of low numbers of pathogens from complex food matrices also impedes their rapid and sensitive detection [31,32]. Antibodies are routinely used as affinity ligands to separate and concentrate the target analyte from sample matrices using paramagnetic beads (PMBs) [31-34] and also as recognition or reporter molecules on immunoassay platforms [31,35,36]. The PMB-captured cells may be presumptively identified by plating them on selective or differential media [37], or their identity may be confirmed by PCR [38,39], flow cytometry [40], or cytotoxicity assay [41]. The use of a biosensor to detect cells captured by immunomagnetic separation (IMS) is an attractive approach due to increased speed, accuracy, and detection of a low number of targets [34,42,43].

Fiber-optic sensors utilize laser excitation to generate an evanescent wave in order to quantify biomolecules immobilized on an optical waveguide [31,44,45]. A capture antibody is immobilized on the waveguide and a fluorescent (Cyanine 5 or Alexa Fluor 647)-labeled second antibody is used as a reporter for the target analyte. Once the laser beam (635 nm) travels through the optical waveguide, it undergoes total internal reflection, and the fluorophore on the reporter antibody bound to the analyte is excited, thereby generating an evanescent wave. The signal is propagated back up to the fiber and is detected in real time by a fluorometer. This format has been successfully applied to many foodborne microorganisms and toxins, however, the limit of detection largely depends on the antibody and the reagents used [31,44,46-48].

In the present study, monoclonal antibodies (MAbs) against *L. monocytogenes* and *Listeria* spp. were generated, characterized, and employed to concentrate *L. monocytogenes* using PMBs. Finally, MAbs were used on the fiber optic sensor to detect *L. monocytogenes* from inoculated food products (soft cheese and hotdogs). In parallel, qPCR and light-scattering sensor methods were performed to confirm the results.

## Results

### MAb production and characterization by ELISA and Western blotting

We selected 11 stable hybridomas, of which 7 (2F2, 2A2, 3B3, 3B7, 4E8, 2D12, and 4E4) reacted with both rInlA and *L. monocytogenes* cells, and 4 (4E5, 4C1, 2A12, and 3F8) reacted with *L. monocytogenes*, *L. innocua*, and *L. seeligeri*. After another round of screening of MAbs-2D12, -3B7, -4E4, and -3F8 against rInlA or *L. monocytogenes* cells (serotypes 4b, 4a, 1/2a, and 1/2b) by ELISA, we chose MAb-2D12 (subclass IgG2a) and MAb-3F8 (subclass IgM) for future use.

**Figure 1:**
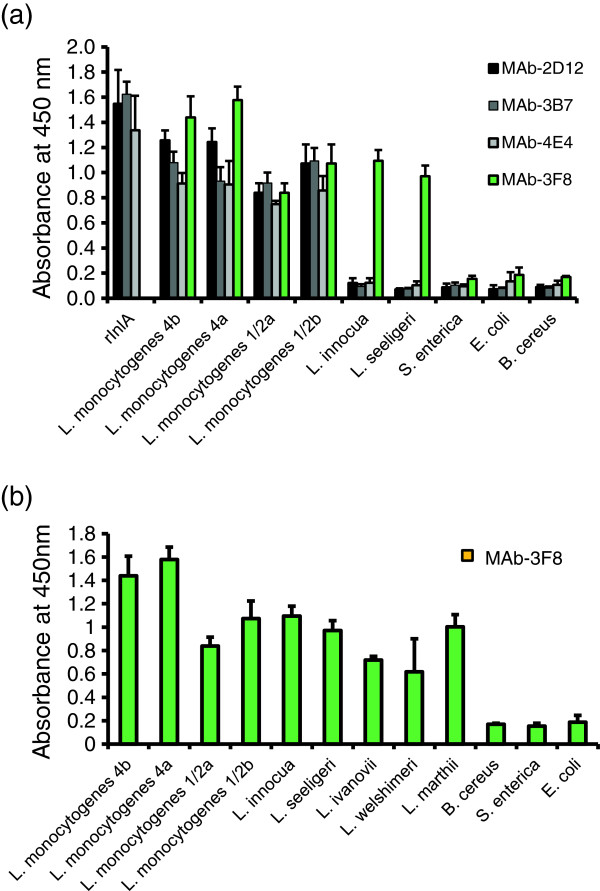
**Indirect ELISA using (a) MAbs 2D12, 3B7, 4E4, and 3F8 or (b) MAb-3F8 against different bacterial strains and purified rInlA.** Several 96-well microtiter plates were coated with live bacteria (~1 × 10^9^ CFU/mL) for 16 h at 4 °C. Data are the mean ± SD of 3 independent assays performed in duplicate.

An ELISA (Figure 1a) revealed that, among the anti-InlA antibodies, MAbs-2D12 and -3B7 strongly reacted (*A*_450_ = 1.0 or higher) with *L. monocytogenes* 4b cells, while MAb-4E4 gave slightly lower reaction values (*A*_450_ = 0.75–0.9). The *Listeria* genus-specific MAb-3F8 gave strong ELISA values (*A*_450_ = 0.8–1.5) when tested against other *Listeria* spp., without producing significant cross-reactions with other bacterial species (Figure 1b).

**Figure 2:**
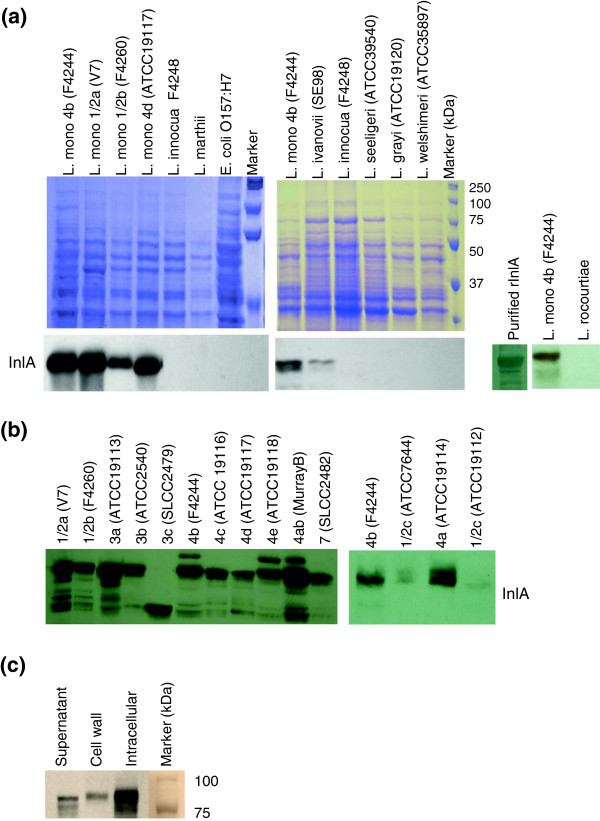
**Western blot analysis showing reaction of anti-InlA antibody (MAb-2D12) to bacterial cell wall proteins.** (**a**) Coomassie blue-stained SDS-PAGE (10 %) gel and corresponding Western blot. (**b**) Western blot with proteins from all 13 serotypes of *L. monocytogenes*. (**c**) Distribution of InlA in cell fractions (4b; F4244): supernatant, cell wall, and intracellular.

In the Western blot, MAb-2D12 reacted with an 80-kDa protein band (InlA) from *L. monocytogenes* and *L. ivanovii*, but it did not react with other *Listeria* spp., including *L. marthii* or *L. rocourtiae* (Figure 2a). MAb-2D12 was reactive with all 13 serotypes; however, a relatively weak reaction with 2 strains of serotype 1/2c (ATCC 19112 and ATCC 7644) was observed. MAb-2D12 also reacted with a 66-kDa band from serotype 3c (SLCC 2479), which is presumably a truncated InlA-protein variant (Figure 2b) [49]. MAb-2D12-reactive InlA was distributed in the secreted, cell wall, and intracellular protein fractions of bacteria (Figure 2c). Immunofluorescence microscopy confirmed the specific binding of anti-InlA antibody (MAb-2D12) to the surface of *L. monocytogenes* cells, but it did not react with *L. innocua* (Additional file 1: Figure S1).

**Figure 3:**
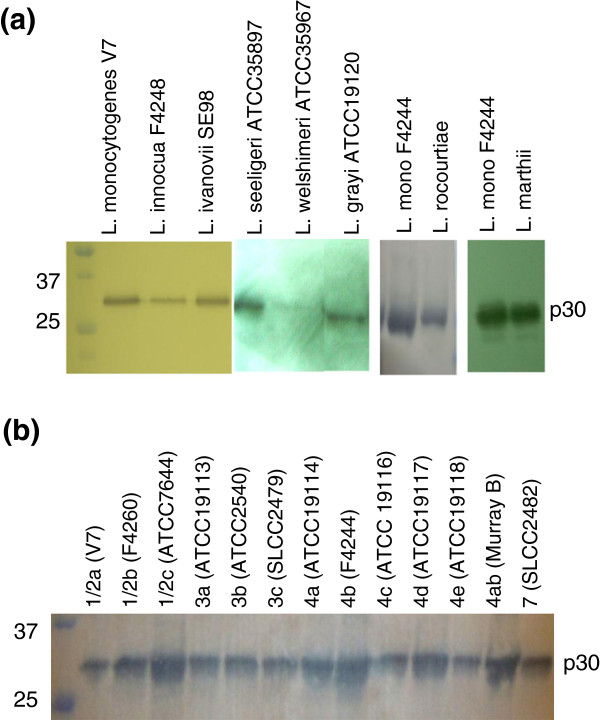
**Western blot showing reaction of MAb-3F8 with cell wall proteins from (a) *Listeria *spp. and (b) serotypes of *L. monocytogenes *.** Proteins were resolved by SDS-PAGE (15 %) before immunoblotting. MAb-3F8 reactive protein (p30) is a 30-kDa protein present in all *Listeria* spp.

MAb-3F8 showed a strong reaction with a single protein band of ~30 kDa (p30) from all *Listeria* spp. with the exception of *L. welshimeri* (Figure 3a). In addition, this MAb showed strong reactions with protein preparations from all 13 serotypes of *L. monocytogenes* (Figure 3b).

### Bacterial capture using antibody-coated paramagnetic beads (PMBs)

**Figure 4:**
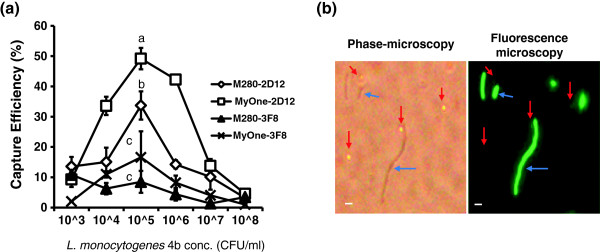
**(a) Capture efficiency of MAb-coated paramagnetic beads from a cell suspension containing variable concentrations of *L. monocytogenes *.** Data are the mean ± SD of three independent assays performed in duplicate. (**b**) Photomicrograph showing capture of GFP-expressing *L. monocytogenes* using MyOne-2D12 (anti-InlA MAb). Beads, red arrow; bacteria, blue arrow; bar = 1 μm.

PMBs with MAb-2D12 had higher capture efficiency than those with MAb-3F8. Using the same antibody, the smaller-sized (1-μm) MyOne beads displayed significantly higher capture efficiency than the Dynabeads M-280 (2.8 μm) for *L. monocytogenes* 4b (F4244) and *L. ivanovii* (ATCC19119) (Table 1, Figure 4). The capture efficiency curve with different concentrations of *L. monocytogenes* cells (10^3^–10^8^ CFU/mL) was bell-shaped; the highest capture (peak) was obtained at 10^5^ CFU/mL, while the lowest capture was obtained at concentrations of 10^3^ CFU/mL and at 10^7^–10^8^ CFU/mL (Figure 4). At initial *L. monocytogenes* concentrations of 10^4^, 10^5^, and 10^6^ CFU/mL, MyOne-2D12 captured 33.5%, 49.2%, and 42.3% of cells, respectively, while M-280-2D12 captured 15%, 33.7%, and 14.2%, respectively. These values were significantly different (*P* < 0.05) from MAb-3F8 conjugated to MyOne or M-280 (Table 1). A similar trend was seen for *L. ivanovii*, but the values obtained were lower than those for *L. monocytogenes*. Therefore, the capture efficiency depends on antibody performance, bead size, and initial bacterial concentration.

**Table 1:** **Immunomagnetic bead-based capture of *Listeria *cells**^**a**^

**Bacteria**	**Concentration (CFU/ml)**	**Percent captured bacteria ± SD**			
		**M-280 (MAb-2D12)**	**MyOne (MAb-2D12)**	**M-280 (MAb-3F8)**	**MyOne (MAb-3F8)**
***L. monocytogenes *F4244**	10^3^	13.5 ± 3.2^Aa^	9.3 ± 2.5^Aa^	10.8 ± 2.9^Aa^	2.0 ± 0.0^Bb^
	10^4^	15.1 ± 4.7^Aa^	33.6 ± 3.0^Cc^	6.35 ± 1.9^Bb^	11.0 ± 1.0^Aa^
	10^5^	33.7 ± 4.7^Cc^	49.2 ± 3.5^Dd^	8.5 ± 3.6^Aa^	16.6 ± 8.6^Aa^
	10^6^	14.3 ± 1.3^Aa^	42.3 ± 1.5^Dd^	4.4 ± 2.1^Bb^	8.2 ± 2.4^Aa^
	10^7^	10.1 ± 4.2^Aa^	13.8 ± 2.3^Aa^	1.3 ± 0^Bb^	4.0 ± 0.3^Bb^
	10^8^	3.2 ± 1.4^Bb^	4.5 ± 0.9^Bb^	3.5 ± 0.6^Bb^	1.0 ± 0.2^Bb^
***L. ivanovii *SE98**	10^3^	5.1 ± 1.1^Bb^	2.0 ± 1.4 ^Bb^	3.8 ± 1.4^Bb^	2.0 ± 1.4^Bb^
	10^4^	3.8 ± 0.8^Bb^	16.4 ± 7.6^Aa^	3.4 ± 1.5^Bb^	7.3 ± 1.5^Bb^
	10^5^	8.8 ± 4.8^Aa^	32.2 ± 3.6^Cc^	2.6 ± 0.5^Bb^	11.2 ± 5.8^Aa^
	10^6^	9.0 ± 1.9^Aa^	34.6 ± 5.6^Cc^	3.8 ± 0.7^Bb^	6.1 ± 1.1^Bb^
	10^7^	5.2 ± 3.4^Bb^	10.0 ± 1.1^Aa^	1.1 ± 0.3^Bb^	2.6 ± 0.7^Bb^
	10^8^	2.8 ± 0.4^Bb^	2.1 ± 0.4^Bb^	2.1 ± 0.7^Bb^	1.5 ± 0.5^Bb^
***L. innocua *F4248**	10^3^	2.0 ± 1.0^Bb^	1.5 ± 0.7^Bb^	2.4 ± 1.2^Bb^	3.5 ± 0.7^Bb^
	10^4^	1.7 ± 0.6^Bb^	2.7 ± 0.5^Bb^	13.3 ± 4.4^Aa^	10.8 ± 2.3^Aa^
	10^5^	1.3 ± 0.2^Bb^	2.4 ± 1.5^Bb^	8.7 ± 0.8^Aa^	14.2 ± 1.6^Aa^
	10^6^	0.2 ± 0.1^Bb^	0.7 ± 0.6^Bb^	3.2 ± 1.9^Bb^	9.0 ± 2.3^Aa^
	10^7^	0.3 ± 0.3^Bb^	0.8 ± 0.6^Bb^	3.0 ± 2.4^Bb^	6.1 ± 2.3^Bb^
	10^8^	0.01 ± 0.0^Bb^	0.2 ± 0.1^Bb^	2.6 ± 2.6^Bb^	1.0 ± 0.2^Bb^
***L. marthii*** BAA-1595	10^3^	2.3 ± 0.5^Bb^	2.0 ± 0.4^Bb^	2.2 ± 0.0^Bb^	4.5 ± 0.7^Bb^
	10^4^	1.5 ± 0.2^Bb^	0.6 ± 0.3^Bb^	4.0 ± 0.8^Bb^	7.7 ± 5.6^Aa^
	10^5^	0.5 ± 0.0^Bb^	2.0 ± 0.4^Bb^	5.3 ± 1.1^Bb^	18.0 ± 3.6^Aa^
	10^6^	0.6 ± 0.1^Bb^	1.3 ± 0.7^Bb^	7.3 ± 1.1^Aa^	5.5 ± 3.0^Bb^
	10^7^	0.2 ± 0.8^Bb^	0.3 ± 0.2^Bb^	2.5 ± 1.8^Bb^	3.2 ± 0.5^Bb^
	10^8^	2.8 ± 0.4^Bb^	0.02 ± 0.0^Bb^	1.1 ± 0.3^Bb^	2.0 ± 0.3^Bb^

^a^Bacteria were grown in TSB-YE for 18 h at 37 °C. The data are average of 3 experiments analyzed in duplicate. Values labeled with different letters (A, B, C, D or a, b, c, d) in a row or in a column are significantly different at *P* < 0.05.

**Figure 5:**
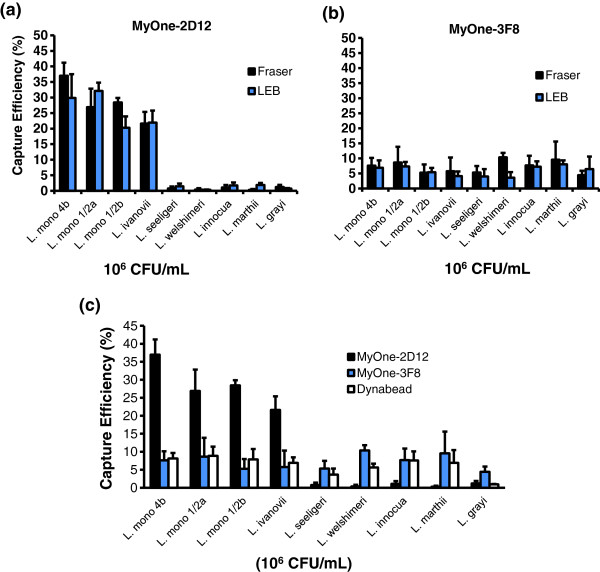
**Capture efficiency and specificity of (a) MyOne-2D12 (InlA); (b) MyOne-3F8 (p30); and (c) MyOne-2D12 (InlA), MyOne-3F8 (p30), and Dynabeads anti- *Listeria *(Dynal).** Bacteria were grown in FB or LEB, and the capture efficiency was determined using a bacterial concentration of ~10^6^ CFU/mL. Data are the mean ± SD of three independent experiments.

All subsequent IMS experiments were performed using MyOne beads. The fluorescence microscopic image in Figure 4b shows the capture of *L. monocytogenes* by MyOne-2D12. The capture efficiency of MyOne-2D12 and MyOne-3F8 was evaluated with bacteria grown in the recommended enrichment broths, LEB or FB. MyOne-2D12 showed significantly higher (*P* < 0.05) capture of *L. monocytogenes* and *L. ivanovii* than other *Listeria* spp., and the capture efficiency was similar for LEB or FB (Figure 5). The capture efficiency for MyOne-2D12 was comparable for the *L. monocytogenes* serotypes tested, including 4b (36.9%), 1/2a (27%), and 1/2b (28%), as well as for a strain of *L. ivanovii* (21.6%), and negligible capture of other *Listeria* spp. was observed (Figure 5a). MyOne-3F8 displayed similar capture efficiency for all *Listeria* spp. tested, irrespective of the enrichment broths used (Figure 5b). When the capture efficiency of MyOne-2D12, MyOne-3F8, and Dynabeads anti-*Listeria* was compared against a *Listeria* panel, MyOne-2D12 captured the most pathogenic *Listeria*. For all other *Listeria* spp., both MyOne-3F8 and Dynabeads anti-*Listeria* had similar values (Figure 5c). Thus, MyOne-2D12 is highly specific for the capture of pathogenic *Listeria*, and MyOne-3F8 and Dynabeads anti-*Listeria* displayed similar capture efficiency for all *Listeria* spp. tested.

**Figure 6:**
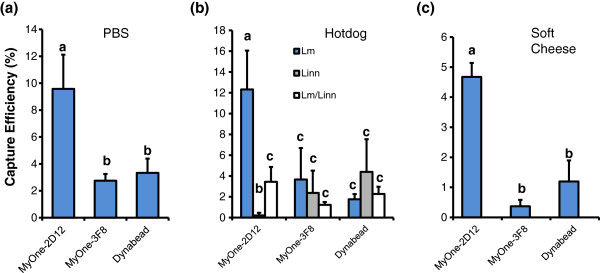
**(a) Capture efficiency of MyOne-2D12 (InlA), MyOne-3F8 (p30), and Dynabeads anti- *Listeria *from a mixed culture of *L. monocytogenes *and *L. innocua *in PBS.** Data are the mean ± SD of three independent assays ± SD. Samples were validated by BARDOT. (**b**) Capture efficiency of PMBs from hotdogs inoculated with *L. monocytogenes* (Lm) and *L. innocua* (Linn) and enriched in FB. (**c**) Capture efficiency of PMB from soft cheese inoculated with *L. monocytogenes* and *L. innocua* and enriched in FB. Samples (b,c) were validated by both BARDOT and real-time qPCR. Capture efficiency (%) are the mean of three independent assays performed in duplicate.

The capture efficiency of PMBs for *L. monocytogenes* in a co-culture with *L. innocua* was also determined (Figure 6). The bacteria were grown in FB, mixed (1:1; 100 μL) in PBS to achieve concentrations of ~1 × 10^5^ CFU/mL each and the capture efficiency was determined by plating followed by BARDOT-based colony identification. MyOne-2D12 captured ~10^4^ CFU/mL (9.5%) of bacteria, of which most colonies (~80%) were confirmed to be *L. monocytogenes* by BARDOT (Figure 6a, Additional file 2: Figure S2). MyOne-3F8 captured ~2.1 × 10^3^ cells (2.75%), and ~50% were confirmed to be *L. monocytogenes*. Dynabeads anti-*Listeria* captured ~2.9 × 10^3^ CFU/mL (3.3%), of which 40% were *L. monocytogenes*.

We also investigated the capture efficiency of bacteria from inoculated food matrices. Hotdogs were inoculated with ~10 CFU/g each of *L. monocytogenes* 4b and *L. innocua* as a mono- or co-culture and enriched for 18 h at 37°C. MyOne-2D12 showed higher capture of *L. monocytogenes* (12%) than *L. innocua* (1%) in the monocultures, but in the co-culture experiment the total bacterial capture dropped to 3.5%. MyOne-3F8 captured 3.7% of the *L. monocytogenes* cells in the monoculture experiment, while the commercial Dynabeads anti-*Listeria* captured only 1.8% (Figure 6b). Dynabeads anti-*Listeria* also captured a numerically (not statistically) higher percentage of *L. innocua* (4.2%) compared with *L. monocytogenes* (1.8%) (Figure 6b). Overall, these data show that MyOne-2D12 captured 10-fold more *L. monocytogenes* than *L. innocua*, while MyOne-3F8 captured 1.5-fold more *L. monocytogenes* than *L. innocua.* Dynabeads anti-*Listeria* had the highest capture efficiency for *L. innocua* from hotdogs.

The capture of *Listeria* was also investigated with soft cheese made from goat’s milk in a co-culture experiment (Figure 6c; Additional file 2: Figure S2). Cheese samples were inoculated with *L. monocytogenes* 4b (~27 CFU/g) and *L. innocua* (32 CFU/g) and enriched in FB for 18 h until the total count reached ~1.7 × 10^8^ CFU/mL. The bacterial capture using MyOne-2D12 was 4.67 ± 0.46%, while MyOne-3F8 (0.37%) and Dynabeads anti-*Listeria* (1.2%) showed significantly (*P* < 0.05) lower capture efficiency (Figure 6c and Additional file 2: Figure S2a). Capture of *L. monocytogenes* colonies on BHI agar plates was verified by a light-scattering sensor, with *L. monocytogenes* and *L. innocua* producing distinct scatter patterns (Additional file 2: Figure S2b).

### Specificity and limit of detection of the fiber-optic sensor

**Figure 7:**
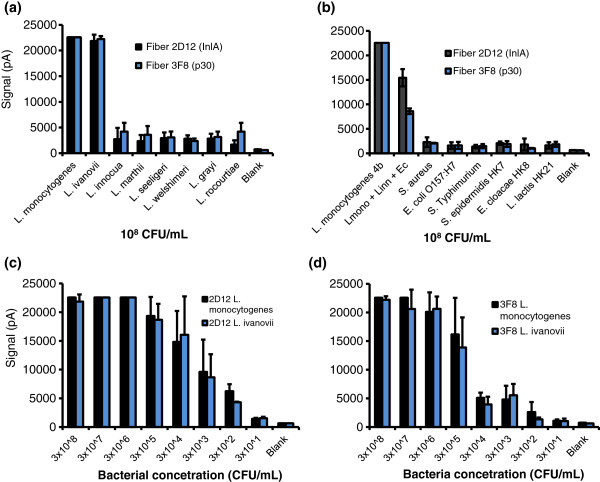
**Determination of specificity (a, b) and detection limit (c, d) of the fiber-optic sensor using MAb-2D12 (InlA) or MAb-3F8 (p30) as capture antibody and Cy5-conjugated anti-InlA MAb-2D12 as a reporter against (a) *Listeria *spp. and (b) other bacteria.** Culture concentrations were 10^8^ CFU/mL (or ~10^6^ CFU/mL for mixed-culture experiments). Detection limit of the fiber-optic sensor using (**c**) MAb-2D12 and (**d**) MAb-3F8 as capture and MAb-2D12 as a reporter against different concentrations of *L. monocytogenes* or *L. ivanovii*. Signals (pA) are the mean of three fibers at 30 s.

The specificity and limit of detection (LOD) of the fiber optic sensor were analyzed by using MAb-2D12 as capture antibody and Cy5-labeled MAb-2D12 as a reporter. The sensor generated strong signals against *L. monocytogenes* and *L. ivanovii*, with a maximum signal of 22,560 pA. In contrast, non-pathogenic *Listeria* produced a maximum signal of 3,000–4,200 pA (Figure 7a), and non-*Listeria* bacteria, including *Salmonella* Typhimurium; *E. coli* O157:H7; and background food contaminant isolates, *Staphylococcus aureus*, *S. epidermidis*, *Enterobacter cloacae*, and *Lactococcus lactis*[50], produced signals of ~2,500 pA (Figure 7b). Similar results were obtained when MAb-3F8 was used as the capture and MAb-2D12 as the reporter molecule (Figure 7a,b). In the mixed cultures containing *L. monocytogenes*, *L. innocua*, and *E. coli* O157:H7 (~10^6^ CFU/mL of each), the signals for MAb-2D12 and MAb-3F8 were 15,440 ± 1,764 pA and 8,440 ± 569 pA, respectively, which were significantly (*P* < 0.05) higher than the values obtained for *L. innocua* (2,725 ± 2,227 pA) or *E. coli* (1,589 ± 662 pA) alone (Figure 7b). The background control (PBS only) values ranged from 504– 650 pA. Therefore, both fiber-optic sensor configurations, 2D12–2D12 and 3F8–2D12, are highly specific for pathogenic *Listeria*, and specificity was contributed primarily by anti-InlA MAb-2D12. Other combinations did not produce satisfactory results (data not shown).

The LOD was also evaluated by using pure cultures of *L. monocytogenes* and *L. ivanovii* serially diluted in PBS (Figure 7c and 7d). Using MAb-2D12 as the capture molecule, the signals increased proportionately as the bacterial concentration increased until a cell concentration of 1 × 10^6^ CFU/mL was reached, which gave the maximum signal (22,560 pA), almost reaching the threshold of the Analyte 2000 fluorometer. The lowest cell concentration that was considered positive (within the detection limit) was 3 × 10^2^ CFU/mL for *L. monocytogenes* (6,252 ± 1,213 pA) and 1 × 10^3^ CFU/mL for *L. ivanovii* (8,657 ± 4,019 pA). These values were at least 2-fold higher than those produced by the samples with 10^1^ cells or PBS (blank). When MAb-3F8 was used as capture antibody, the LOD for *L. monocytogenes* (16,156 ± 6,382 pA) and *L. ivanovii* (13,882 ± 5,250 pA) was ~1 × 10^5^ CFU/mL (Figure 7d).

### IMS coupled with a fiber-optic sensor for detection of *L. monocytogenes*

**Figure 8:**
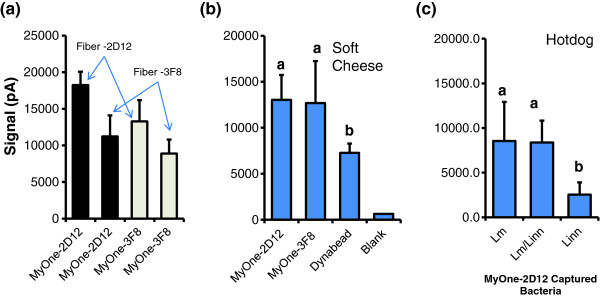
**Fiber-optic-based detection of *L. monocytogenes *after immunomagnetic capture with MyOne-2D12 or MyOne-3F8 from (a) buffer, (b) soft cheese, or (c) hotdog samples.** (**a**) Fibers were coated with MAb-2D12 and 3F8. (**b**, **c**) Fibers were coated with MAb-2D12 only. Cy5-conjugated MAb-2D12 was used as a reporter in all experiments. Data (signals; pA) are the mean of 3 fibers. Bars marked with different letters are significantly different (*P* < 0.05). Blank, PBS only.

Bacteria captured by MyOne-2D12 or MyOne-3F8 were detected by the MAb-2D12-coated fiber-optic sensor (with MAb-2D12 as a reporter) and yielded signals of 18,230 ± 1,840 pA and 13,280 ± 2,890 pA, respectively (Figure 8). The MAb-3F8 fiber optic sensor (with MAb-2D12 as a reporter) produced signals of 11,225 ± 2,860 pA and 8,890 ± 1,900 pA, respectively (Figure 8a). The fiber optic signal value for MyOne-2D12 and -3F8 captured *L. monocytogenes* was about 2 to 3-fold higher than the signals obtained from the LOD concentrations (3 × 10^2^ CFU/ml) (Figure 7). These data indicate that *L. monocytogenes* detection using MAb-2D12 for IMS and a fiber optic sensor gave better results compared with those obtained using MAb-3F8.

In soft cheese-containing co-culture of *L. monocytogenes* and *L. innocua*, both MyOne-2D12 and MyOne-3F8 captured bacteria and produced signals of 13,026 ± 2,710 pA and 12,620 ± 4,554 pA, respectively (Figure 8b). Bacteria captured with Dynabeads anti-*Listeria* gave the lowest fiber-optic signals (Figure 8b). In *Listeria*-inoculated hotdog samples, only MyOne-2D12 was used for IMS and assayed by fiber optic sensor. The signal from the sample containing both *L. monocytogenes* and *L. innocua* was 8,376 ± 2,448 pA, while that from *L. monocytogenes*- and *L. innocua*-inoculated food was 8,552 ± 4,363 pA and 2,549 ± 1,358 pA, respectively (Figure 8c). For both food samples, the fiber optic signal values for MyOne-2D12 and -3F8 captured *L. monocytogenes* but not the *L. innocua* were higher than the signals obtained from the LOD cell concentrations (3 × 10^2^ CFU/ml) (Figure 7). Therefore, the IMS and fiber optic sensor can be used together for detection of *L. monocytogenes* from enriched food samples, even in presence of *L. innocua* or other bacteria.

### Real-time qPCR for validation

Real-time qPCR targeting *hly*A was used to quantify PMB-captured *Listeria* from hotdogs and goat’s cheese artificially contaminated with *L. monocytogenes* and *L. innocua* (Table 2). When IMS was applied to the cheese samples followed by qPCR, MyOne-2D12 showed cell counts that were 4 times higher than those of MyOne-3F8 and Dynabeads anti-*Listeria*. In hotdog samples, MyOne-2D12 produced cell counts that were 2–3 times higher than those of the other 2 types of beads.

**Table 2:** **qPCR analysis of paramagnetic bead captured bacteria from food samples**^**a**^

**Paramagnetic bead (PMB)**	**Detection/enumeration of PMB captured cells by qPCR (CFU/ml)**			
	**Hotdog**^**b**^		**Soft Cheese**^**b**^	
	**CFU/ml ± SD**	**%**	**CFU/ml ± SD**	**%**
MyOne-2D12	1.09 ± 3.07 × 10^7^	12.62 ± 3.5^A^	2.65 ± 1.79 × 10^7^	16.2 ± 9.7^A^
MyOne-3F8	2.26 ± 1.18 × 10^6^	2.63 ± 1.4^B^	6.45 ± 7.44 × 10^6^	3.8 ± 4.3^B^
Dynabead anti-*Listeria*	2.76 ± 3.11 × 10^6^	6.12 ± 0.5^B^	7.65 ± 8.26 × 10^6^	4.4 ± 4.8^B^

^a^qPCR analysis is based on *hlyA*. Primers to 16S gene sequences were used as internal control.

^b^Data are average of 3 experiments run in triplicate. Values labeled with letters (A, B) in a column are significantly different at *P* < 0.05.

## Discussion

The recovery of low numbers of target pathogens from complex food matrices is a challenge for sensitive detection methods [31,32]. IMS using PMBs is used to separate and concentrate target pathogens from food samples before detection by plating, immunoassay, PCR, or biosensor methods [31,37,39,42,45,51]. Antibodies [14] or alternative molecules [19,51,52] are used as capture molecules for IMS, and improvements in reagents and assay platform development are essential to enhance assay performance.

The specific detection of whole cells of *L. monocytogenes* using immunological methods relies on highly specific antibodies with a strong affinity for bacterial surface antigens [31]. The antigen target should be uniformly distributed on the target organism, covalently anchored to the cell wall, and accessible to the antibody [53]. InlA is a well-characterized protein that is highly specific to *L. monocytogenes* and *L. ivanovii*, and it has all the desirable properties of an antigen [15]. Thus, we produced MAbs against InlA (pathogenic *Listeria*) and p30 (all *Listeria* spp.). The resulting MAbs were employed in IMS to capture and concentrate bacteria from food followed by fiber-optic sensor-based detection. To the best of our knowledge, this is the first demonstration of the combined use of these two approaches.

InlA-specific antibody production was facilitated by the use of whole cells of *L. monocytogenes* and purified rInlA as immunogens. Hybrid B-lymphocyte clones secreted antibodies with a strong reaction towards live whole cells, but a weaker reaction was observed with heat-killed cells (data not shown). Since rInlA was soluble, denaturing agents were not required before immunization. Thus, the native structure of InlA during the immune response was preserved, and the resulting antibody recognized the native protein on the surface of bacteria. The InlA-specific MAb-2D12 reacted with all known *L. monocytogenes* serotypes, whereas previously reported MAbs failed to recognize all 13 serotypes [23,26,27]. Only serotype 1/2c showed a weak reaction with MAb-2D12. However, this strain has been involved in a few sporadic cases of listeriosis [54,55] and is rarely found. Moreover, none of the 25 strains of serotype 1/2c expressed a functional, full-length InlA [55], which may explain why MAb-2D12 displayed a reduced reaction to 1/2c. When tested with serotype 3c, MAb-2D12 reacted strongly with a ~66 kDa band instead of the normal 80-kDa InlA band. The smaller band may represent truncated InlA, which results from *inlA* mutation [49]. Generally, such strains are less invasive and are less likely to cause systemic infection as confirmed in animal models [56].

We also generated a *Listeria* species-specific MAb by immunization with whole cells of *L. monocytogenes*. MAb-3F8 (IgM subclass) reacted with a ~30-kDa protein (p30) present in all eight *Listeria* species. Therefore, MAb-3F8 may aid tracking of *Listeria* contamination in foods or the food-production environment.

The separation of target organisms following primary enrichment using IMS is faster than using selective secondary enrichment [57]. Thus, we performed IMS using two different sizes of commercial beads. Antibody-coated 1-μm MyOne T1 exhibited significantly higher capture efficiency than the 2.8-μm M-280 beads (Table 1, Figure 4). Similarly, Foddai et al. [58] used six different magnetic beads, including the two types used in this study, to capture *Mycobacterium avium*. MyOne displayed better capture efficiency than that of M-280, but the overall capture efficiency was low (<10%). In the present study, the capture efficiency for MyOne-2D12 and M280-2D12 was 49.2% and 33.7% (initial concentration used, 10^5^ CFU/mL), respectively while 16.6% for MyOne-3F8 and 8.5% for M280-3F8. Paoli et al. [52] used M-280-coated scFv antibody to ActA and reported a maximum capture of 19% for *L. monocytogenes*. Walcher et al. [51] reported a capture range of 46%–122% using a bacteriophage endolysin specific for *Listeria* spp. coated on M-280; however, the long capture incubation time (2 h) may have allowed bacterial growth, thereby producing a higher capture rate. Furthermore, the binding of bacteriophage to host cells is an irreversible process, which may lead to higher capture efficiency than with antibody-coated PMBs. Koo et al. [19] used Hsp60-coated M-280, which showed a capture efficiency for *L. monocytogenes* of 1.8%–9.2%.

The capture efficiency also depended on the initial bacterial concentration. The highest capture (peak) with MyOne-2D12 or MyOne-3F8 was seen at a bacterial concentration of 10^5^ CFU/mL (Figure 4). This is important for meaningful comparisons to be made between the performances of IMS in different studies, which may use a wide range of initial bacterial concentrations. Collectively, IMS data indicate that beads with a smaller diameter (1-μm MyOne) have better capture efficiency than larger beads (2.8-μm M-280) due to higher surface area to mass ratio and smaller beads can bind more antibody per mg of beads (20 μg biotinylated antibody for MyOne vs. 10 μg for M-280) (Invitrogen). Furthermore, the antibody affinity, the distribution/expression of antigens on the surface of bacteria, and the initial bacterial concentration also significantly affect capture efficiency [14,58]. Here, the abundant expression of InlA on the surface of *L. monocytogenes* cells coupled with the use of smaller sized PMB was most likely responsible for increased capture efficiency. However, the assay performance may be affected if PMB followed by fiber optic sensor was applied to food samples directly without an enrichment step. In such situation, food matrices may affect bacterial antigen expression or antibody affinity [14].

We tested the capture efficiency of *L. monocytogenes* in a co-culture experiment in buffer or food. Food contaminated with *L. monocytogenes* may contain other *Listeria* spp. and background competitive microflora [16,50]. *L. monocytogenes* grows slowly and is a poor competitor; hence, lower cell numbers are expected in food samples [18]. In a mixed population, *L. monocytogenes* may be outgrown by other species of *Listeria* during enrichment [17,18,21,33]. Here, IMS using MyOne-2D12 efficiently captured *L. monocytogenes*, in the presence of *L. innocua* while both MyOne-3F8 and Dynabeads anti-*Listeria* captured more *L. innocua* cells than *L. monocytogenes* (Figure 6). Furthermore, the capture efficiency for MyOne-2D12 using a co-culture in buf-fer or food varied from 4.7%–12.3% (Figure 6 and Additional file 2: Figure S2). Less than optimal level of capture was attributed largely to the presence of higher initial concentrations of bacteria (10^7^–10^8^ CFU/mL) in the sample and the presence of interfering agents (inhibitors) in food matrices, particularly in soft cheese. Furthermore, the increased capture of *L. monocytogenes* in hotdog compared to PBS was possibly due to increased expression of MAb-2D12-reactive antigen (InlA) during enrichment while cells used in PBS were originally cultured in BHI, which may have caused reduced InlA expression resulting in reduced *L. monocytogenes* capture (Figure 6).

*L. ivanovii* is an opportunistic human pathogen that is associated with gastroenteritis and bacteremia in humans [13,59]; therefore, the development of methods to detect this pathogen is also essential. MAb-2D12 reacted with *L. ivanovii*, which was successfully detected by using IMS and a fiber-optic sensor. Hearty et al. [60] reported the InlA-specific MAb-2B3; however, this antibody was unable to detect *L. ivanovii* in their assay setup. MAb-2B3 may be specific for an epitope of InlA on *L. monocytogenes* that is absent on *L. ivanovii*.

PMB-captured cells were also identified by BARDOT and qPCR. BARDOT is a light-scattering sensor that detects and identifies bacterial colonies on agar plates with a high degree of precision in minutes, since each species has a distinctive scatter-fingerprint signature [61]. BARDOT allowed quantitative estimation of capture rate for *L. monocytogenes* and *L. innocua* on BHI or MOX plates (Additional file 2: Figure S2) instantly based on colony scatter patterns and it is easy to perform without the requirement for any additional reagents or probes.

Real-time qPCR confirmed that *L. monocytogenes* capture and detection from food by MyOne-2D12 was 13%–16%, which is significantly higher than that by MyOne-3F8 and Dynabeads anti-Listeria (3%–6%). These estimations are slightly higher than the plate count and the light-scattering data obtained in this study. Yang et al. [39] used nanoparticles for IMS and showed better capture and detection of *L. monocytogenes* in milk with real-time PCR (9%) compared with plate counts (6%). This may be because qPCR detects DNA from nonviable or viable but non-culturable cells, which may not otherwise be detected by traditional plating methods [62,63].

The fiber-optic sensor operates based on the principles of antibody-antigen interaction and is marketed by Research International. It is currently used for foodborne or biothreat agent detection [31]. The antibody (MAb-2D12) used in this study on the optical waveguide made the assay highly specific for *L. monocytogenes* and *L. ivanovii*, with the detection limit of 3 × 10^2^ CFU/ml, a significant improvement over previous reports. Geng et al. [46] used MAb-C11E9 to show cross-reaction with some *L. innocua* strains with LOD of 4.3 × 10^3^ CFU/ml. Using a polyclonal anti-*Listeria* capture antibody and an InlA-specific aptamer as a reporter, Ohk et al. [48] reported specific detection of *L. monocytogenes* with a LOD of 10^3^ CFU/mL.

## Conclusions

We developed highly specific anti-InlA MAb (2D12) against pathogenic *Listeria*: *L. monocytogenes* and *L. ivanovii* and anti-p30 MAb (3F8) against all *Listeria* spp. including the two new species (*L. marthii* and *L. rocourtiae*). Anti-InlA antibody allowed specific detection of low levels (3 × 10^2^ CFU/ml) of *L. monocytogenes* and *L. ivanovii* when used on IMS and a fiber-optic sensor in the presence of other bacteria from buffer, soft cheese or hotdogs inoculated with low levels of cells (10–40 CFU/g) following enrichment.

## Methods

### Culture and growth conditions

All bacterial cultures (Additional file 3: Table S1) were maintained on brain heart infusion (BHI; Acumedia, Lansing, MI) agar plates at 4°C with the exception of lactic acid bacteria, which were maintained on de Man Rogosa Sharpe agar (MRS; Becton Dickinson [BD], Sparks, MD). To obtain fresh cultures, *Listeria* spp. were grown in tryptic soy broth (TSB; BD) containing 0.6% yeast extract (TSB-YE) or *Listeria* enrichment broth (LEB; BD) at 37°C for 16–18 h. Non-*Listeria* organisms were grown in TSB-YE, and lactic acid bacteria were grown in MRS broth at 37°C for 16–18 h. Fraser Broth (FB) and modified Oxford agar (MOX) were purchased from BD. All bacteria were maintained in BHI broth with 20% glycerol at −80°C until further use.

### Cloning of *inlA* and immunogen preparation

Specific primers (MWG-Biotech, Huntsville, AL) were designed to target the *inlA* gene (GenBank acc. no.: DQ132795) using Vector NTI 10.0 software (Invitrogen) in order to amplify the complete open reading frame (2331 bp) except for the signal peptide and a C-terminal portion. To insert the *inl*A gene into the pAE expression vector [64], the restriction sites for *Bam*HI and *Kpn*I enzymes were incorporated into the forward primer, For-inlA (5′-CGGGATCCGTATGGATTAACACGA-3′) and reverse primer, Rev-inlA (5′-GGGGTACCCTAAGTAAGAACCATTGCAGT-3), respectively. The *inl*A ORF was amplified from the genomic DNA of *L. monocytogenes* (ATCC 19114) by PCR using an Eppendorf thermocycler (Mastercycler EP gradient S) with the following standardized conditions 94°C for 7 min, 94°C for 1 min, 45°C for 1 min, 68°C for 2 min, and a final extension of 68°C for 7 min. The amplicon was digested with *Bam*HI and *Kpn*I and ligated into pAE—predigested with the same enzymes—using T4 DNA Ligase (Invitrogen). The pAE-*inl*A construct was electrotransformed into *Escherichia coli* Top10 (Invitrogen), the recombinant clones were selected on LB agar containing ampicillin (100 μg/mL), and insertion of *inlA* (pAE-*inlA*) was confirmed by sequencing. The pAE-*inlA* plasmid was transformed into *E. coli* BL21(DE3) pLysS (Invitrogen) competent cells. The transformed cells were grown to reach the log phase (OD_600_ = 0.5–0.7) and induced with 1 mM IPTG for 3 h at 37°C. Cells were harvested, suspended in lysis buffer (100 mM NaH_2_PO_4_, 10 mM Tris HCl, and 20 mM imidazole; pH 8.0) and sonicated (3 cycles using a Branson Sonifier). The recombinant InlA (rInlA) containing a poly-histidine tag (6×-His) was purified by using a Ni-NTA affinity chromatography system (GE Healthcare, Piscataway, NJ). Finally, column-eluted proteins were dialyzed against 0.02 M phosphate buffered saline (PBS; pH 7.2) for 24 h and concentrated with polyethylene glycol (MW 20,000).

### Immunization, MAb production, and MAb characterization

Six-week-old BALB/c female mice were administered intraperitoneally (i.p.) with approximately 1 × 10^8^ cells/mL of heat-killed *L. monocytogenes* serotype 4b diluted in PBS and mixed (1:1) with complete Freund’s adjuvant (CFA). Two weeks later, a mixture of heat-killed *L. monocytogenes* and 50 μg of rInlA prepared with incomplete Freund’s adjuvant (IFA) was administered i.p. every week for 8 weeks. Four days before the last immunization, the mouse showing the highest antibody titer against rInlA in an indirect ELISA received booster immunizations with rInlA via both intravenous and i.p. routes. The splenocytes were harvested from the mouse and fused with murine Sp2/O-Ag14 myeloma cells in the presence of 50% (w/v) PEG 1450 (Sigma) as described previously [65]. Selected hybridoma clones were administered to pristane-primed mice to produce ascitic fluid for antibody production [65](28). MAbs were purified by affinity chromatography using a protein A-Sepharose 4B column (GE Healthcare), and the class and subclass of each MAb were determined by ELISA using a Mouse Subisotyping Kit (Sigma).

Indirect ELISA was performed to determine the reactivities of MAbs with live bacterial cultures adjusted to OD_600_ = 1 (approx. 10^9^ CFU/mL) in 0.1 M sodium carbonate coating buffer (pH 9.6) or with rInlA (10 ng/well) for 16 h at 4°C, and immunoassay was carried out as described previously [24].

### Protein preparation, SDS-PAGE, and Western blot

Bacterial proteins were prepared according to the published method [66] with some modifications. For isolation of cell wall-associated proteins, 100 mL of cultures grown for 18 h were centrifuged (7000 × *g* for 10 min), and the cell pellets were resuspended by gently pipetting up and down with 250 μL of protein extraction buffer (0.5% SDS, 10 mM Tris; pH 6.9) followed by incubation at 37°C for 30 min. After centrifugation (16,100 × *g* for 10 min at 4°C), the supernatants were collected. The remaining cell pellets were resuspended in sample solvent (4.6% SDS, 10% β-mercaptoethanol, 0.124 M Tris, and 20% glycerol; pH 6.9), sonicated four times for 15 s each (Branson Sonifier), and centrifuged (16100 × *g* for 20 min at 4°C) to collect the supernatant (representing intracellular protein fractions). Protein concentrations were adjusted using the bicinchoninic acid assay (BCA; Pierce) and separated by SDS-PAGE (10% or 12% acrylamide; Bio-Rad, Hercules, CA). The proteins were blotted onto Immobilon-P membranes (Millipore, Bedford, MA) and blocked with 5% skimmed milk for 1 h at room temperature (RT). The membranes were washed with PBST (PBS containing 0.05% Triton X-100), immunoprobed sequentially with the MAbs, and incubated with HRP-conjugated goat anti-mouse polyvalent antibody (Sigma). Antibody-reactive bands were visualized following treatment with a chemiluminescence substrate system (ECL kit; Thermo Fisher Scientific, Rockford, IL) or DAB (6 mg of 3.3′-diaminobenzidine tetrahydrochloride; 10 μL of H_2_O_2_, 30%; 9 mL of 50 mM Tris–HCl, pH 7.6; 1 mL of 0.3% NiCl_2_). Two MAb-producing clones were selected for further study: *L. monocytogenes* (InlA-reactive)-specific MAb-2D12 and *Listeria* genus-specific (p30-reactive) MAb-3F8.

### Immunofluorescence microscopy

*L. monocytogenes* (serotypes 4b, 1/2a, 1/2b, and 4d) and *L. innocua* cell pellets (grown in 10 mL of LEB) were washed twice with PBS and resuspended in 1 mL of PBS containing 5% bovine serum albumin (PBS-BSA). Subsequently, 20 μL of cells were incubated with MAbs diluted in 500 μL PBS-BSA for 1 h at 37°C. After washing with PBS (2×), the cell pellets were resuspended in 250 μL of FITC-conjugated goat anti-mouse IgG (1:100; Sigma) and incubated at 37°C for 1 h. After three sequential washes with PBS, the pellets were stained with Hoechst 33258 (for nuclear staining) for 15 min, and a single drop of the suspension was examined using an epifluorescence microscope (Leica, Buffalo Grove, IL).

### Antibody labeling

For use with a fiber-optic sensor and magnetic beads that are pre-coated with streptavidin, affinity-purified antibodies were biotinylated using the EZ-Link Sulfo NHS-Biotinylation Kit (Pierce) as per the manufacturer’s instructions. The biotinylated MAbs were tested by ELISA in avidin-coated microtiter plates, and the ratio of biotin incorporated into the MAbs was calculated using the HABA assay (4′-hydroxyazoben-zene-2-carboxylic acid; Pierce). For use with a fiber-optic sensor, MAbs were also labeled with Cy5 using the Cy5-Ab labeling kit (Amersham Biosciences) as per the manufacturer’s protocol.

### Bacterial capture using antibody-coated PMBs

Two different sizes of PMBs were used: Dynabeads M-280 Streptavidin (2.8-μm diameter) and MyOne streptavidin T1 (1.0 μm-diameter) (Invitrogen). Bead preparation involved mixing the streptavidin-coupled PMBs with 200 μg/mL of biotinylated MAbs for 30 min under constant rotation at RT. The unbound biotinylated MAbs were separated by removing the PMBs with a magnetic particle concentrator (MPC-S; Invitrogen), followed by washing the beads three times with PBS containing 1% BSA. The beads were stored at 4°C until use.

To determine PMB-based capture with pure cultures, bacterial cultures grown for 18 h were washed twice with PBS and resuspended in PBS containing 0.1% BSA. Subsequently, 20 μL of MAb-coated PMBs was added to 200 μL of bacterial cell suspension containing variable cell counts (10^3^ to 10^8^ CFU/mL) and mixed in a rotary incubator for 30 min at RT. PMBs were recovered using MPC-S, washed 3 times using 1 mL of PBST, and resuspended in 200 μL of PBS. Finally, PMBs were subjected to vigorous vortexing to release the captured bacteria and 100 μL of each suspension was surface-plated onto BHI or MOX agar plates for enumeration [19]. In some experiments, Dynabeads Anti-*Listeria* (Invitrogen) were used in parallel as a control. The capture efficiency (CE) was calculated as follows: CE (%) = Cb/Ci × 100, where Cb is number of cells bound to beads (CFU/mL) and Ci is the initial total number of cells present in the sample (CFU/mL).

To verify PMB-based capture of *Listeria* from food matrices, we inoculated 10 g of each RTE soft cheese made from goat’s milk and hotdogs (purchased from local grocery stores in West Lafayette, IN) with *L. monocytogenes* and *L. innocua* (10–40 CFU/g) and incubated the samples for 15 min at 25°C. The samples were placed in stomacher bags built with an interior filter lining (Whirl-Pak; Nasco, Fort Atkinson, WI) and 90 mL of FB or LEB was added to each bag, blended for 2 min in a stomacher, and incubated at 37°C for 18 h. Uninoculated food samples served as negative controls. A total of 10 mL of each enriched culture was placed in a 15-mL tube, washed twice with PBST, and resuspended in 10 mL of PBST. Samples were diluted 10-fold in PBS, and IMS was performed as described above using 200 μL of the inoculated sample. The precise levels of inoculums and growth after enrichment were enumerated on BHI and MOX agar after 24 h or 48 h, respectively, at 37°C. Bead-captured bacteria were further tested by fiber-optic sensor, light-scattering sensor, and qPCR.

### Fiber-optic immunosensor assay

Polystyrene waveguides (fibers) were cleaned and coated with 100 μg/mL of streptavidin (NeutrAvidin; Pierce) for 2 h at 4°C as described previously [48]. Fibers were blocked with SuperBlock blocking buffer (Pierce) for 1 h and incubated overnight at 4°C with each of the biotinylated MAbs (200 μg/mL). The fibers were rinsed gently with PBST and then reacted with biotinylated-BSA (100 μg/mL; Pierce) for 1 h at RT to block unbound streptavidin sites. The antibody coated fibers could be stored at 4°C until use. The fibers were washed again in PBST and placed in reaction chambers containing 100 μL of freshly harvested bacterial suspensions (Table 1) at various concentrations (1 × 10^3^ to 1 × 10^8^ CFU/mL) and incubated for 2 h at RT. Following gentle washing with PBS, the fibers were exposed to Cy5-labeled anti-InlA antibody for 2 h at 4°C, washed with PBST, and signals were acquired with an Analyte 2000 Fluorometer (Research International Co., Monroe, WA). The fluorescence intensity signals were recorded for each fiber for 30 s [46]. For each treatment, 3–5 waveguides were used, and mean values ± SD for each experiment were presented.

### Confirmation of captured bacteria using an optical light-scattering sensor

An automated light-scattering sensor, BARDOT (BActerial Rapid Detection using Optical light-scattering Technology; Advanced Bioimaging Systems, LLC, West Lafayette, IN) was used to identify colonies of *Listeria* captured by IMS (described above) on BHI or MOX agar plates [19,61]. This system collects scatter images of bacterial colonies (diameter, 1.3 ± 0.2 mm) through a diode laser (635 nm), and the bacteria were identified by comparing scatter images with library-stored images [61]. Before conducting the food sample testing experiment, initial experiments were performed to determine the capture rate of IMS for *L. monocytogenes* and *L. innocua*, present at 10^6^ CFU/mL each in a mixture in PBS, followed by BARDOT analysis.

### Real-time quantitative PCR (qPCR)

PMB-captured bacteria were also analyzed by qPCR. To eliminate PCR inhibitors, the DNA was purified from captured bacteria using the DNeasy Blood and Tissue Kit (Qiagen) by treating the PMB–bacteria complexes (100 μL) with 180 μL lysis buffer (20 mM Tris–HCl, pH 8.0; 2 mM sodium EDTA; 1.2% Triton X-100; 20 mg/mL lysozyme) followed by incubation at 37°C for 30 min. PMBs were removed from the solutions by using MPC-S (Invitrogen), and the supernatant was pipetted onto the columns. DNA was eluted in 100 μL of elution buffer and used for qPCR.

Primers specific for *hly*A (hlyA-For, 5′-TGCAAGTCCTAAGACGCCA-3′ and hlyA-Rev, 5′-CACTGCATCTCCGTGGTATACTAA-3′) of *L. monocytogenes* were used for detection [67]. Primers for 16 s (Lis-16 s-For, 5′- CACGTGGGCAACCTGCCTGT-3′ and Lis-16 s-Rev, 5′- CTAATGCACCGCGGGCCCAT-3′) were used as an internal control. The qPCR was performed using Power SYBR Green Master Mix (Applied Biosystems, Foster City, CA) with 5 μL of DNA template in a 20-μL total reaction volume and analyzed in triplicate. PCR amplification was carried out in a StepOnePlus Real-Time PCR System (Applied Biosystems) under the following conditions: 1 cycle of 95°C for 10 min for denaturation, followed by 40 cycles of 95°C for 20 s, 58°C for 1 min, and 95°C for 1 min for the dissociation curve. To construct the standard curves, DNA from *L. monocytogenes* F4244 was quantified, and a serial dilution was prepared to produce a concentration curve. In all qPCR assays, the DNA templates of *L. monocytogenes* and *L. innocua* were used as internal controls. Bacterial cell counts were estimated based on the Ct values of unknown samples and compared with the standard curve [39].

### Statistical analysis

Data are expressed as the mean ± SD from at least three independent experiments performed in duplicate unless otherwise indicated. Mean values were compared by ANOVA using GraphPad Prism version 5.0 (GraphPad Software), and the differences in mean values were compared using Tukey’s multiple comparison test at *P* < 0.05.

## Competing interests

The authors declare that no competing interests exist.

## Authors' contributions

This project was conceived and designed by MM, FRC, WPS, JAGA, AKB; experiments were performed by MM, NLC, ANM; data were analyzed by MM, JAGA, AKB; and written by MM, JAGA and AKB. Graduate work of MM was supervised by JAGA and AKB. All authors read and approved the final manuscript.

## Supplementary Material

Additional file 1 **Figure S1.** Indirect immunofluorescence assay of *L. monocytogenes* (top row) and *L. innocua* (bottom row) immunoprobed with anti-InlA MAb-2D12 and FITC-conjugated anti-mouse antibodies. Cells were counter-stained with Hoechst for nuclear staining to assess the total bacterial cells. Magnification, 1000×. Click here for file

Additional file 2 **Figure S2.** Capture efficiency of MyOne-2D12 (InlA), MyOne-3F8 (p30), and Dynabeads anti-*Listeria* (Dynal) from soft cheese inoculated with *L. monocytogenes* and *L. innocua* and enriched in FB. Captured cells were plated on (a) MOX plates for enumeration and (b) BHI for confirmation of *L. monocytogenes* (Lm) and *L. innocua* (Linn) counts by a light-scattering sensor, BARDOT. Click here for file

Additional file 3 **Table S1.** Description of bacterial strains used. Click here for file

## References

[B1] Vazquez-Boland JA Kuhn M Berche P Chakraborty T Dominguez-Bernal G Goebel W Gonzalez-Zorn B Wehland J Kreft J Listeria pathogenesis and molecular virulence determinants Clin Microbiol Rev 2001 14 3 584 640 10.1128/CMR.14.3.584-640.2001 11432815 PMC88991

[B2] Azevedo I Regalo M Mena C Almeida G Carneiro L Teixeira P Hogg T Gibbs P Incidence of Listeria spp. in domestic refrigerators in Portugal Food Control 2003 16 2 121 124

[B3] von Laer AE Lima ASL Trindade PS Andriguetto C Destro MT Silva WP Characterization of Listeria monocytogenes isolated from a fresh mixed sausage processing line in Pelotas-RS by PFGE Braz J Microbiol 2009 40 574 582 10.1590/S1517-83822009000300021 PMC3768533 24031402

[B4] Delgado da Silva MC Destro MT Hofer E Tibana A Characterization and evaluation of some virulence markers of Listeria monocytogenes strains isolated from Brazilian cheeses using molecular, biochemical and serotyping techniques Int J Food Microbiol 2001 63 3 275 280 10.1016/S0168-1605(00)00426-8 11246911

[B5] Pintado CM Grant KA Halford-Maw R Hampton MD Ferreira MA McLauchlin J Association between a case study of asymptomatic ovine listerial mastitis and the contamination of soft cheese and cheese processing environment with Listeria monocytogenes in Portugal Foodborne Pathog Dis 2009 6 5 569 575 10.1089/fpd.2008.0246 19388828

[B6] Olsen SJ Patrick M Hunter SB Reddy V Kornstein L MacKenzie WR Lane K Bidol S Stoltman GA Frye DM Multistate outbreak of Listeria monocytogenes infection linked to delicatessen turkey meat Clin Infect Dis 2005 40 7 962 967 10.1086/428575 15824987

[B7] Miya S Takahashi H Ishikawa T Fujii T Kimura B Risk of Listeria monocytogenes contamination of raw ready-to-eat seafood products available at retail outlets in Japan Appl Environ Microbiol 2010 76 10 3383 3386 10.1128/AEM.01456-09 20348310 PMC2869148

[B8] CDC Multistate outbreak of Listeriosis associated with Jensen Farms cantaloupe - United States, August-September 2011 MMWR Morb Mortal Wkly Rep 2011 60 39 1357 1358 21976119

[B9] Scallan E Hoekstra RM Angulo FJ Tauxe RV Widdowson MA Roy SL Jones JL Griffin PM Foodborne illness acquired in the United States–major pathogens Emerg Infect Dis 2011 17 1 7 15 21192848 10.3201/eid1701.P11101 PMC3375761

[B10] FAO/WHO Food and Agriculture Organization World Health Organization. Risk assessment of *Listeria monocytogenesin* ready to eat foods-Technical report 2004 1 267 vol.5;

[B11] Graves LM Helsel LO Steigerwalt AG Morey RE Daneshvar MI Roof SE Orsi RH Fortes ED Milillo SR den Bakker HC Listeria marthii sp. nov., isolated from the natural environment, Finger Lakes National Forest Int J Syst Evol Microbiol 2010 60 Pt 6 1280 1288 19667380 10.1099/ijs.0.014118-0

[B12] Leclercq A Clermont D Bizet C Grimont PA Le Fleche-Mateos A Roche SM Buchrieser C Cadet-Daniel V Le MA Lecuit M Listeria rocourtiae sp. nov Int J Syst Evol Microbiol 2010 60 Pt 9 2210 2214 19915117 10.1099/ijs.0.017376-0

[B13] Guillet C Join-Lambert O Le MA Leclercq A Mechai F Mamzer-Bruneel MF Bielecka MK Scortti M Disson O Berche P Human listeriosis caused by Listeria ivanovii Emerg Infect Dis 2010 16 1 136 138 10.3201/eid1601.091155 20031061 PMC2874378

[B14] Banada PP Bhunia AK Zourob M, Elwary S, Turner A Antibodies and immunoassays for detection of bacterial pathogens Principles of Bacterial Detection: Biosensors, Recognition Receptors and Microsystems 2008 Manchester: Cambridge University 567 602

[B15] Bierne H Cossart P Listeria monocytogenes surface proteins: from genome predictions to function Microbiol Mol Biol Rev 2007 71 2 377 397 10.1128/MMBR.00039-06 17554049 PMC1899877

[B16] O'Connor L O'Leary M Leonard N Godinho M O'Reilly C Coffey L Egan J O'Mahony R The characterization of Listeria spp. isolated from food products and the food-processing environment Lett Appl Microbiol 2010 51 5 490 498 10.1111/j.1472-765X.2010.02928.x 20831655

[B17] Oravcova K Trncikova T Kuchta T Kaclikova E Limitation in the detection of Listeria monocytogenes in food in the presence of competing Listeria innocua J Appl Microbiol 2008 104 2 429 437 17887983 10.1111/j.1365-2672.2007.03554.x

[B18] Besse NG Barre L Buhariwalla C Vignaud ML Khamissi E Decourseulles E Nirsimloo M Chelly M Kalmokoff M The overgrowth of Listeria monocytogenes by other Listeria spp. in food samples undergoing enrichment cultivation has a nutritional basis Int J Food Microbiol 2010 136 3 345 351 10.1016/j.ijfoodmicro.2009.10.025 19945759

[B19] Koo OK Aroonnual A Bhunia AK Human heat-shock protein 60 receptor-coated paramagnetic beads show improved capture of Listeria monocytogenes in the presence of other Listeria in food J Appl Microbiol 2011 111 1 93 104 10.1111/j.1365-2672.2011.05040.x 21535331

[B20] Meldrum RJ Ellis PW Mannion PT Halstead D Garside J Prevalence of Listeria monocytogenes in ready-to-eat foods sampled from the point of sale in Wales, United Kingdom J Food Prot 2010 73 8 1515 1518 20819364 10.4315/0362-028x-73.8.1515

[B21] Carvalheira A Eus bio C Silva J Gibbs P Teixeira P Influence of L. innocua on the growth of L. monocytogenes Food Control 2010 21 11 1492 1406 10.1016/j.foodcont.2010.04.021

[B22] Byrne B Stack E Gilmartin N Kennedy RO Antibody-based sensors: Principles, problems and potential for detection of pathogens and associated toxins Sensors 2009 9 6 4407 4445 10.3390/s90604407 22408533 PMC3291918

[B23] Bhunia AK Johnson MG Monoclonal antibody specific for Listeria monocytogenes associated with a 66-kilodalton cell surface antigen Appl Environ Microbiol 1992 58 6 1924 1929 1622267 10.1128/aem.58.6.1924-1929.1992 PMC195705

[B24] Bhunia AK Ball PH Fuad AT Kurz BW Emerson JW Johnson MG Development and characterization of a monoclonal antibody specific for Listeria monocytogenes and Listeria innocua Infect Immun 1991 59 9 3176 3184 1715321 10.1128/iai.59.9.3176-3184.1991 PMC258150

[B25] Kim SH Park MK Kim JY Chuong PD Lee YS Yoon BS Hwang KK Lim YK Development of a sandwich ELISA for the detection of Listeria spp. using specific flagella antibodies J Vet Sci 2005 6 1 41 46 15785122

[B26] Heo SA Nannapaneni R Story RP Johnson MG Characterization of new hybridoma clones producing monoclonal antibodies reactive against both live and heat-killed Listeria monocytogenes J Food Sci 2007 72 1 M008 M015 10.1111/j.1750-3841.2006.00202.x 17995886

[B27] Lin M Armstrong S Ronholm J Dan H Auclair ME Zhang Z Cao X Screening and characterization of monoclonal antibodies to the surface antigens of Listeria monocytogenes serotype 4b J Appl Microbiol 2009 106 5 1705 1714 10.1111/j.1365-2672.2008.04140.x 19226395

[B28] Paoli GC Chen CY Brewster JD Single-chain Fv antibody with specificity for Listeria monocytogenes J Immunol Methods 2004 289 1–2 147 155 15251420 10.1016/j.jim.2004.04.001

[B29] Lathrop AA Banada PP Bhunia AK Differential expression of InlB and ActA in Listeria monocytogenes in selective and nonselective enrichment broths J Appl Microbiol 2008 104 627 639 10.1111/j.1365-2672.2007.03574.x 17927754

[B30] Nannapaneni R Story R Bhunia AK Johnson MG Unstable expression and thermal instability of a species-specific cell surface epitope associated with a 66-kilodalton antigen recognized by monoclonal antibody EM-7 G1 within serotypes of Listeria monocytogenes grown in nonselective and selective broths Appl Environ Microbiol 1998 64 8 3070 3074 9687476 10.1128/aem.64.8.3070-3074.1998 PMC106818

[B31] Bhunia AK Biosensors and bio-based methods for the separation and detection of foodborne pathogens Adv Food Nutr Res 2008 54 1 44 18291303 10.1016/S1043-4526(07)00001-0

[B32] Brehm-Stecher B Young C Jaykus L-A Tortorello ML Sample preparation: The forgotten beginning J Food Protect 2009 72 1774 1789 10.4315/0362-028x-72.8.1774 19722419

[B33] Gasanov U Hughes D Hansbro PM Methods for the isolation and identification of Listeria spp. and Listeria monocytogenes: a review FEMS Microbiol Rev 2005 29 5 851 875 10.1016/j.femsre.2004.12.002 16219509

[B34] Tu SI Reed S Gehring A He YP Simultaneous detection of Escherichia coli O157:H7 and Salmonella Typhimurium: The use of magnetic beads conjugated with multiple capture antibodies Food Anal Methods 2011 4 3 357 364 10.1007/s12161-010-9175-z

[B35] Dwivedi HP Jaykus L-A Detection of pathogens in foods: the current state-of-the-art and future directions Cri Rev Microbiol 2011 37 1 40 63 10.3109/1040841X.2010.506430 20925593

[B36] Velusamy V Arshak K Korostynska O Oliwa K Adley C An overview of foodborne pathogen detection: In the perspective of biosensors Biotechnol Adv 2010 28 2 232 254 10.1016/j.biotechadv.2009.12.004 20006978

[B37] Wadud S Leon-Velarde CG Larson N Odumeru JA Evaluation of immunomagnetic separation in combination with ALOA Listeria chromogenic agar for the isolation and identification of Listeria monocytogenes in ready-to-eat foods J Microbiol Methods 2010 81 2 153 159 10.1016/j.mimet.2010.02.014 20211665

[B38] Bilir Ormanci FS Erol I Ayaz ND Iseri O Sariguzel D Immunomagnetic separation and PCR detection of Listeria monocytogenes in turkey meat and antibiotic resistance of the isolates Br Poult Sci 2008 49 5 560 565 10.1080/00071660802298328 18836902

[B39] Yang H Qu L Wimbrow AN Jiang X Sun Y Rapid detection of Listeria monocytogenes by nanoparticle-based immunomagnetic separation and real-time PCR Int J Food Microbiol 2007 118 2 132 138 10.1016/j.ijfoodmicro.2007.06.019 17716768

[B40] Hibi K Abe A Ohashi E Mitsubayashi K Ushio H Hayashi T Ren H Endo H Combination of immunomagnetic separation with flow cytometry for detection of Listeria monocytogenes Anal Chim Acta 2006 573-574 158 163 17723519 10.1016/j.aca.2006.03.022

[B41] Gray KM Bhunia AK Specific detection of cytopathogenic Listeria monocytogenes using a two-step method of immunoseparation and cytotoxicity analysis J Microbiol Methods 2005 60 2 259 268 10.1016/j.mimet.2004.10.006 15590100

[B42] Gehring A Tu SI High-throughput biosensors for multiplexed food-borne pathogen detection Annu Rev Anal Chem 2011 4 151 172 10.1146/annurev-anchem-061010-114010 21689045

[B43] Koo OK Liu Y Shuaib S Bhattacharya S Ladisch MR Bashir R Bhunia AK Targeted capture of pathogenic bacteria using a mammalian cell receptor coupled with dielectrophoresis on a biochip Anal Chem 2009 81 8 3094 3101 10.1021/ac9000833 19317455

[B44] Leung A Shankar PM Mutharasan R A review of fiber-optic biosensors Sens Actuat B: Chem 2007 125 2 688 703 10.1016/j.snb.2007.03.010

[B45] Taitt CR Anderson GP Ligler FS Evanescent wave fluorescence biosensors Biosens Bioelectron 2005 20 12 2470 2487 10.1016/j.bios.2004.10.026 15854820

[B46] Geng T Morgan MT Bhunia AK Detection of low levels of Listeria monocytogenes cells by using a fiber-optic immunosensor Appl Environ Microbiol 2004 70 10 6138 6146 10.1128/AEM.70.10.6138-6146.2004 15466560 PMC522132

[B47] Lim DV Simpson JM Kearns EA Kramer MF Current and developing technologies for monitoring agents of bioterrorism and biowarfare Clin Microbiol Rev 2005 18 4 583 607 10.1128/CMR.18.4.583-607.2005 16223949 PMC1265906

[B48] Ohk SH Koo OK Sen T Yamamoto CM Bhunia AK Antibody-aptamer functionalized fibre-optic biosensor for specific detection of Listeria monocytogenes from food J Appl Microbiol 2010 109 3 808 817 10.1111/j.1365-2672.2010.04709.x 20337767

[B49] Olier M Pierre F Rousseaux S Lemaitre JP Rousset A Piveteau P Guzzo J Expression of truncated Internalin A is involved in impaired internalization of some Listeria monocytogenes isolates carried asymptomatically by humans Infect Immun 2003 71 3 1217 1224 10.1128/IAI.71.3.1217-1224.2003 12595435 PMC148840

[B50] Kim H Bhunia AK SEL, a selective enrichment broth for simultaneous growth of Salmonella enterica, Escherichia coli O157:H7, and Listeria monocytogenes Appl Environ Microbiol 2008 74 15 4853 4866 10.1128/AEM.02756-07 18539786 PMC2519329

[B51] Walcher G Stessl B Wagner M Eichenseher F Loessner MJ Hein I Evaluation of paramagnetic beads coated with recombinant Listeria phage endolysine derived cell-wall-binding domain proteins for separation of Listeria monocytogenes from raw milk in combination with culture-based and real-time polymerase chain reaction based quantification Foodborne Pathog Dis 2010 7 9 1019 1024 10.1089/fpd.2009.0475 20500083

[B52] Paoli GC Kleina LG Brewster JD Development of Listeria monocytogenes-specific immunomagnetic beads using a single-chain antibody fragment Foodborne Pathog Dis 2007 4 1 74 83 10.1089/fpd.2006.65 17378711

[B53] Tully E Hearty S Leonard P O'Kennedy R The development of rapid fluorescence-based immunoassays, using quantum dot-labeled antibodies for the detection of Listeria monocytogenes cell surface proteins Int J Biol Macromol 2006 39 1–3 127 134 16600361 10.1016/j.ijbiomac.2006.02.023

[B54] Bueno VF Banerjee P Banada PP de Jose MA Lemes-Marques EG Bhunia AK Characterization of Listeria monocytogenes isolates of food and human origins from Brazil using molecular typing procedures and in vitro cell culture assays Int J Environ Health Res 2010 20 1 43 59 10.1080/09603120903281283 20104385

[B55] Jacquet C Doumith M Gordon JI Martin PM Cossart P Lecuit M A molecular marker for evaluating the pathogenic potential of foodborne Listeria monocytogenes J Infect Dis 2004 189 11 2094 2100 10.1086/420853 15143478

[B56] Chen Y Ross WH Whiting RC Van SA Nightingale KK Wiedmann M Scott VN Variation in Listeria monocytogenes dose responses in relation to subtypes encoding a full-length or truncated internalin A Appl Environ Microbiol 2011 77 4 1171 1180 10.1128/AEM.01564-10 21169442 PMC3067222

[B57] Varshney M Yang LJ Su XL Li YB Magnetic nanoparticle-antibody conjugates for the separation of Escherichia coli O157:H7 in ground beef J Food Protect 2005 68 9 1804 1811 10.4315/0362-028x-68.9.1804 16161677

[B58] Foddai A Elliott CT Grant IR Maximizing capture efficiency and specificity of magnetic separation for Mycobacterium avium subsp. paratuberculosis cells Appl Environ Microbiol 2010 76 22 7550 7558 10.1128/AEM.01432-10 20851966 PMC2976214

[B59] Snapir YM Vaisbein E Nassar F Low virulence but potentially fatal outcome - Listeria ivanovii Eur J Intern Med 2006 17 4 286 287 10.1016/j.ejim.2005.12.006 16762780

[B60] Hearty S Leonard P Quinn J O'Kennedy R Production, characterisation and potential application of a novel monoclonal antibody for rapid identification of virulent Listeria monocytogenes J Microbiol Methods 2006 66 2 294 312 10.1016/j.mimet.2005.12.009 16457899

[B61] Banada PP Huff K Bae E Rajwa B Aroonnual A Bayraktar B Adil A Robinson JP Hirleman ED Bhunia AK Label-free detection of multiple bacterial pathogens using light-scattering sensor Biosens Bioelectron 2009 24 6 1685 1692 10.1016/j.bios.2008.08.053 18945607

[B62] Duodu S Mehmeti I Holst-Jensen A Loncarevic S Improved sample preparation for real-time PCR detection of in hot-smoked salmon using filtering and immunomagnetic separation techniques Food Anal Methods 2009 2 23 29 10.1007/s12161-008-9043-2

[B63] Lindback T Rottenberg ME Roche SM Rorvik LM The ability to enter into an avirulent viable but non-culturable (VBNC) form is widespread among Listeria monocytogenes isolates from salmon, patients and environment Vet Res 2010 41 1 8 10.1051/vetres/2009056 19796607 PMC2775167

[B64] Ramos CRR Abreu PAE Nascimento A Ho PL A high-copy T7 Escherichia coli expression vector for the production of recombinant proteins with a minimal N-terminal his-tagged fusion peptide Brazilian J Med Biol Res 2004 37 8 1103 1109 10.1590/s0100-879x2004000800001 15273812

[B65] Harlow E Lane D Antibodies: A Laboratory Manual 1988 NY: Cold Spring Harbor

[B66] Jonquieres R Bierne H Fiedler F Gounon P Cossart P Interaction between the protein InlB of Listeria monocytogenes and lipoteichoic acid: a novel mechanism of protein association at the surface of gram-positive bacteria Mol Microbiol 1999 34 5 902 914 10.1046/j.1365-2958.1999.01652.x 10594817

[B67] Nogva HK Rudi K Naterstad K Holck A Lillehaug D Application of 5'-nuclease PCR for quantitative detection of Listeria monocytogenes in pure cultures, water, skim milk, and unpasteurized whole milk Appl Environ Microbiol 2000 66 10 4266 4271 10.1128/AEM.66.10.4266-4271.2000 11010869 PMC92295

