# Supplementary figures and images for: Highly specific fiber optic immunosensor coupled with immunomagnetic separation for detection of low levels of Listeria monocytogenes and L. ivanovii
Source: BMC Microbiol. 2012 Nov 23;12:275. doi: 10.1186/1471-2180-12-275 (PMC3533925; doi:10.1186/1471-2180-12-275)

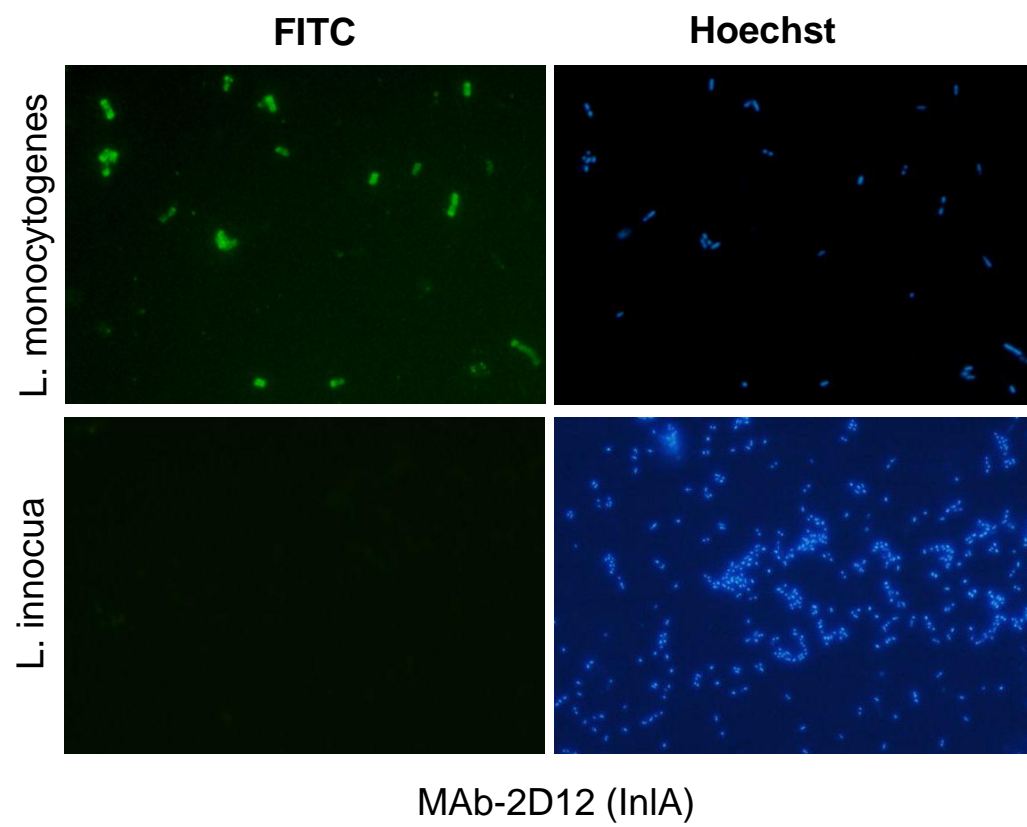

Fig S1

Supplement: Additional file 1 — Figure S1. Indirect immunofluorescence assay of L. monocytogenes (top row) and L. innocua (bottom row) immunoprobed with anti-InlA MAb-2D12 and FITC-conjugated anti-mouse antibodies. Cells were counter-stained with Hoechst for nuclear staining to assess the total bacterial cells. Magnification, 1000×. [file 1471-2180-12-275-S1.pdf]

(a)

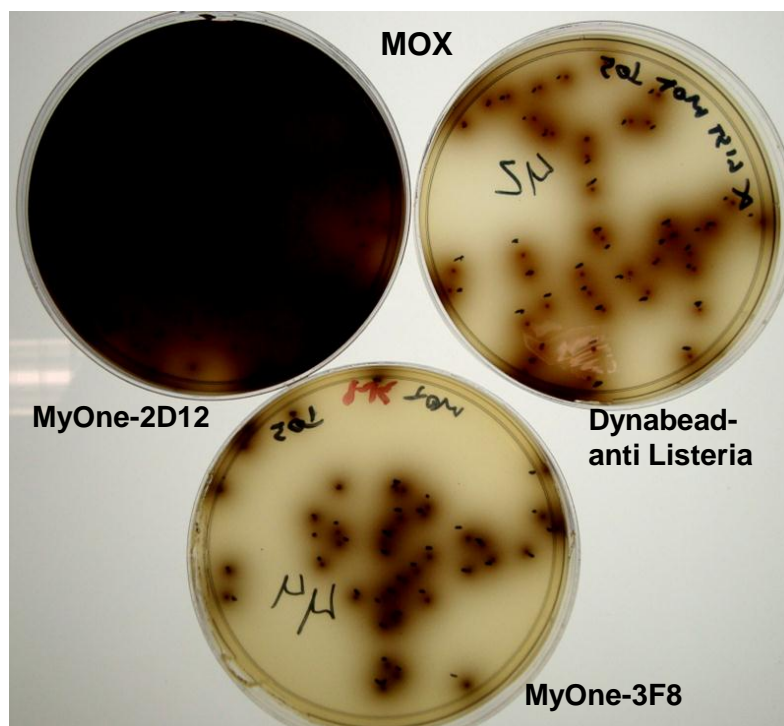

(b)

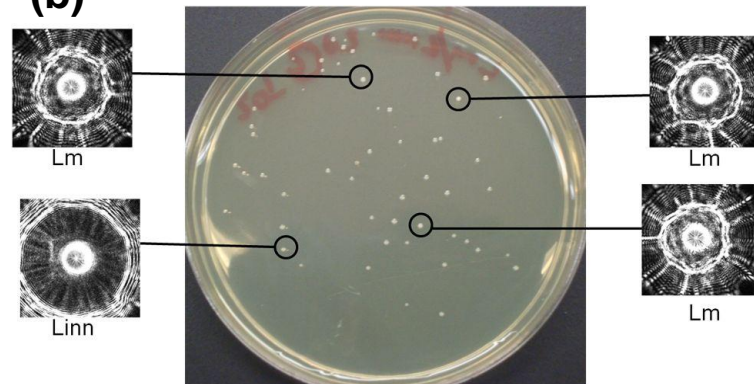

Fig S2

Supplement: Additional file 2 — Figure S2. Capture efficiency of MyOne-2D12 (InlA), MyOne-3F8 (p30), and Dynabeads anti-Listeria (Dynal) from soft cheese inoculated with L. monocytogenes and L. innocua and enriched in FB. Captured cells were plated on (a) MOX plates for enumeration and (b) BHI for confirmation of L. monocytogenes (Lm) and L. innocua (Linn) counts by a light-scattering sensor, BARDOT. [file 1471-2180-12-275-S2.pdf]
