# Supplementary material for: Highly specific fiber optic immunosensor coupled with immunomagnetic separation for detection of low levels of Listeria monocytogenes and L. ivanovii
Source: BMC Microbiol. 2012 Nov 23;12:275. doi: 10.1186/1471-2180-12-275 (PMC3533925; doi:10.1186/1471-2180-12-275)
Supplement: Additional file 3 — Table S1. Description of bacterial strains used. [file 1471-2180-12-275-S3.docx]

**Table S1.** Description of bacterial strains used

| Species | Serotype | Strain / Isolates | Source/Ref*^a^* |
| --- | --- | --- | --- |
| *Listeria monocytogenes* | 1/2a | V7 | FDA |
| *L. monocytogenes* | 1/2b | F4260 | CDC |
| *L. monocytogenes* | 1/2c | 7644 | ATCC |
| *L. monocytogenes* | 4a | 19114 | ATCC |
| *L. monocytogenes* | 4b | F4244 | CDC |
| *L. monocytogenes* | 4b | 19115 | ATCC |
| *L. monocytogenes* | 4c | 19116 | ATCC |
| *L. monocytogenes* | 4d | 19117 | ATCC |
| *L. monocytogenes* | 4e | 19118 | ATCC |
| *L. monocytogenes* | 4ab | Murray B | FDA |
| *L. monocytogenes* | 3a | 19113 | ATCC |
| *L. monocytogenes* | 3b | 2540 | ATCC |
| *L. monocytogenes* | 3c | 2479 | SLCC |
| *L. monocytogenes* | 7 | 2482 | SLCC |
| *L. monocytogenes* | 4b | Scott A | FDA |
| *L. monocytogenes* | 1/2c | 19112 | ATCC |
| *L. innocua* | 6a | 11288 | ATCC |
| *L. innocua* |  | F4248 | CDC |
| *L. innocua* | 6a | Li01 | UFPel |
| *L. welshimeri* |  | 35897 | ATCC |
| *L. seeligeri* |  | 3954 | ATCC |
| *L. seeligeri* |  | Ls02 | UFPel |
| *L. ivanovii* |  | 19119 | ATCC |
| *L. ivanovii* |  | SE98 | USDA |
| *L. grayi* |  | 19120 | ATCC |
| *L. marthii* |  | BAA-1595 | ATCC |
| *L. rocourtiae* |  | CIP 109804 | Ref # 12 |
| *Salmonella enterica* ser. Typhimurium | | DUP-1167 | MFM-Purdue |
| *Salmonella enterica* ser. Enteritidis |  | 13076 | ATCC |
| *Bacillus subtilis* |  | 6633 | ATCC |
| *Bacillus thuringiensis* |  | DUP - 6044 | MFM-Purdue |
| *Escherichia coli* | O157:H7 | EDL933 | CDC |
| *Lactococcus lactis* |  | 11454 | MFM-Purdue |
| *Enterococcus aerogenes* |  | DUP-14591 | MFM-Purdue |
| *Lactobacillus paracasei* |  | DUP-13076 | MFM-Purdue |
| *Klebsiella pneumonia* |  | --- | MFM-Purdue |
| *Enterococcus faecalis* |  | --- | MFM-Purdue |
| *Lactococcus lactis* subsp. *lactis* |  | HK21 | MFM-Purdue |
| *Enterobacter cloacae* |  | HK8 | MFM-Purdue |
| *Staphylococcus epidermidis* |  | HK7 | MFM-Purdue |
| *Bacillus cereus* |  | 11778 | ATCC |
| *Staphylococcus aureus* |  | 13301 | ATCC |
| *Pseudomonas aeruginosa* |  | 10145 | ATCC |

*^a^* FDA: Food and Drug Administration, Washington, D.C.; CDC: Centers for Disease Control and Prevention, Atlanta, GA. ; ATCC: American Type Culture Collection, Rockville, MD.; SLCC: Special Listeria Culture Collection, Institute of Hygiene and Microbiology, Univ. of Würzburg, Germany; USDA: National Center for Agricultural Utilization Research, Peoria, Illinois, U.S.A.; MFM-Purdue: Molecular Food Microbiology Lab. Collection, Purdue.; UFPel: Laboratório de Microbiologia de Alimentos Collection, FAEM-UFPel. *L. rocourtiae* was kindly provided by M. Weidmann and H. Oliver.
